# Thoracic epidural anaesthesia vs intrathecal morphine in dogs undergoing major thoracic and abdominal surgery: clinical study

**DOI:** 10.1186/s12917-022-03296-3

**Published:** 2022-05-27

**Authors:** E. Lardone, D. Sarotti, D. Giacobino, E. Ferraris, P. Franci

**Affiliations:** 1 Department of Veterinary Sciences, School of Veterinary Medicine, University of Turin, 2 Largo Paolo Braccini, 10095 Grugliasco, Italy; 2 Centro Veterinario Fossanese, 29/E Via Cuneo, 12045 Fossano, Italy

**Keywords:** thoracic epidural anaesthesia, intrathecal morphine, dog, major surgery

## Abstract

**Background:**

There is scant clinical research on neuraxial analgesia in dogs undergoing major surgery. With this study we compared the perioperative analgesic effects of thoracic epidural anaesthesia (TEA) and intrathecal morphine (ITM) in dogs scheduled for thoracic or cranial abdominal surgery. The dogs received methadone and dexmedetomidine, were anaesthetized with propofol maintained with sevoflurane, and randomly assigned to receive either TEA (ropivacaine 0.5% at 0.2 mg/kg and morphine 0.1 mg/kg administered at T_12_-T_13_) or ITM (morphine 30 μg/kg administered at L_6_-L_7_). Intraoperative rescue analgesia (iRA) was fentanyl 1 μg/kg administered if heart rate or mean arterial pressure increased by 30% above the pre-stimulation level. Glasgow Pain Composite Scale score (GPCS) dictated the use of postoperative rescue analgesia (pRA) with methadone 0.2 mg/kg.

**Results:**

There was a statistically significant difference in iRA, median time to first fentanyl bolus, median fentanyl dose after surgical opening, and median GPCS score at 30 minutes (min), 1 ,2, 4, 6, and 8 hours (h) between the two groups (*p<*0.001; *p<*0.001; *p*<0.001; *p<*0.01; *p<*0.01; *p<*0.001; *p<*0.01; *p=*0.01; *p=*0.01, respectively). Fewer TEA than ITM group dogs required iRA during surgical opening and pRA: 5% (1/18) and 2/18 (11%), respectively, in the TEA and 83% (16/18) and 10/18 (55%), respectively, in the ITM group. Side effects were urinary retention in 3/18 (16%) TEA group dogs and 2/18 (11%) ITM group dogs and prolonged sedation in 2/18 (11%) in ITM group dogs.

TEA and ITM were effective in managing perioperative pain in dogs undergoing thoracic or cranial abdominal surgery.

**Supplementary Information:**

The online version contains supplementary material available at 10.1186/s12917-022-03296-3.

## Background

One of most challenging tasks for anaesthetists is to ensure effective analgesia during major thoracic or cranial abdomen surgery. Regional anaesthesia plays key role in providing analgesia in human patients undergoing major open surgeries [1].

Two common neuraxial techniques for severe acute postoperative pain in human patients are thoracic epidural analgesia (TEA) and intrathecal morphine (ITM). There is scant clinical research on neuraxial analgesia in dogs undergoing thoracotomy and cranial abdomen surgery, however [2–4]. To our best knowledge, no prospective clinical studies in veterinary anaesthesia have investigated the efficacy of TEA in treating perioperative pain, although the technique for accessing thoracic epidural space in dogs is the same as that described for humans decades ago [5, 6].

Intrathecal morphine has gained wide acceptance for postoperative analgesia, however, the incidence of postoperative nausea and vomiting and pruritus has limited its use in human clinical practice [7]. Though frequently used in labour analgesia, it is still a matter of clinical research and discovery since the first clinical report appeared 120 years ago [8–10].

Intrathecal morphine administered together with local anaesthetics achieves good analgesia in the early postoperative period in dogs undergoing orthopaedic surgery but is burdened by pruritus and urinary retention, the two major unwanted effects [11–13]. Epidural morphine has been studied [2], but data are lacking on intrathecal anaesthesia for perioperative thoracotomy and cranial abdominal surgery analgesia. To fill this gap, we compared intraoperative nociceptive stimuli control, postoperative analgesia, and perioperative side effects after the administration of TEA (single shot injection of ropivacaine and morphine) and intrathecal morphine in dogs undergoing thoracic or cranial abdominal surgery.

## Methods

**Fig. 1:**
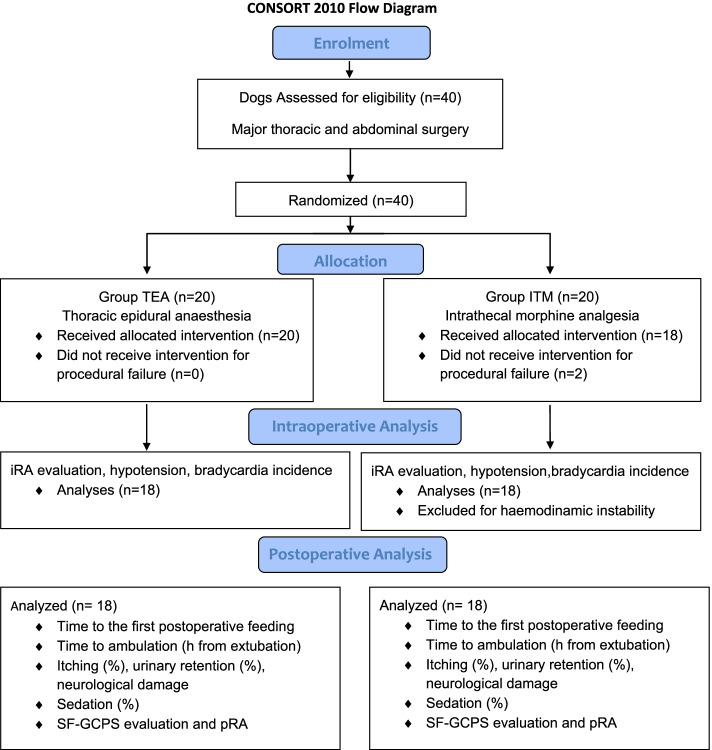
Consolidated standards of reporting trials flow diagram. Intraoperative rescue analgesia (iRA), Short Form Glasgow Composite Pain Scale (SF-GCPS)

This randomized clinical trial was approved by the Ethical Committee of the Department of Veterinary Science of Turin, Italy (n. 71 10/01/2020). The study was performed at the Veterinary Teaching Hospital, University of Turin, between January 2020 and December 2021. Informed owner written consent was obtained for all dogs. The manuscript conforms to the Consolidated Standards of Reporting Trials (CONSORT) Statement 2010 for reporting randomized clinical trials [14] (Fig. 1).

Based on the assumption that the dogs receiving ITM had a higher median Glasgow Composite Pain Scale (GCPS) score than those receiving thoracic epidural anaesthesia at 8 h after extubation, we calculated an effect size of 1 (as suggested by a pilot study), with 18 dogs per group to identify a difference in GCPS score with 90% power and 5% alpha. Accounting for possible dropouts, we decided to enrol 22 dogs per group. The study sample was dogs undergoing lateral or sternal thoracotomy or cranial abdominal surgery involving the abdominal wall only or the abdominal wall and organs, and with American Society of Anesthesiologists (ASA) physical status classification I, II or III. Exclusion criteria were: 1) aggressive behaviour; 2) American College of Veterinary Internal Medicine *(*ACVIM*) s*tage C of the ACVIM staging system for the treatment of heart disease; 4) spinal deformity; 5) infection at the injection site; 6) coagulopathy or conditions contraindicating administration of the study drugs.

The dogs were randomly allocated to one of the two treatment groups (thoracic epidural anaesthesia [TEA] or intrathecal morphine [ITM]) by simple randomization based on a computer-generated randomization sequence (www.randomizer.org). Methadone 0.2 mg/kg (Semfortan; 10 mg/mL, Dechra Veterinary Products, UK) and dexmedetomidine 1 μg/kg (Dexdomitor; 0.5 mg/mL, Orion Pharma, Finland) were administered intramuscularly (IM) into the quadriceps muscle. Anaesthesia was induced approximately 20 min after intravenous (IV) administration of propofol (Proposure, 10 mg/mL, Merial, Italy) to effect via a pre-placed cephalic cannula and maintained with sevoflurane (Sevoflo, Poets, Belgium); the end tidal expired concentration was 1.2% as the starting target delivered in oxygen and medical air (FiO_2_ 0.4) via a circle breathing system after orotracheal intubation. Lactated Ringer’s solution (lactated Ringer’s; Fresenius Kabi, Italy) was administered at 5 mL/kg/h as starting infusion rate until surgical access to the thoracic or the abdominal cavity was achieved. Depth of anaesthesia was clinically assessed monitoring eye position, palpebral reflex, and jaw tone. In the event of intraoperative movement or presence of a brisk palpebral reflex associated with a nociceptive response to surgery, a propofol bolus dose of 1 mg/kg was delivered at the anaesthetist’s discretion. Volume-controlled ventilation (Primus, Draeger, Germany) was imposed; the settings were adjusted to maintain an end-expiratory carbon dioxide pressure (PE’CO2) between 35 and 45 mmHg (4.6-6 kPa). Heart rate (HR), invasive systolic, mean, and diastolic arterial blood pressure (SAP, MAP, DAP), respiratory rate (RR), end tidal isoflurane fraction (FE’Iso), PE’CO2, and haemoglobin oxygen saturation were monitored (Infinity Delta and Primus, Draeger) and manually recorded every 5 min. A dorsal-pedal artery was catheterized to monitor arterial blood pressure. Hair clipping was performed as two small patches from the spinous process of T11 to L1 and from L4 to L7.

### Epidural anaesthesia

The clipped area of the skin was aseptically prepared with chlorhexidine (4%) and alcohol (70%). Epidural anaesthesia was administered using a Tuohy needle (Perican 22, 20 or 18 G; B. Braun, Germany) with the dog in lateral recumbency on the side to be operated on, the spinal column kept gently flexed and the dorsal part of the animal body positioned beyond the edge of the operating table. With index and thumb of one hand leant respectively on the spinous and the articular process of T13, the needle was inserted via paramedian approach on the dependent side, lateral to the spinal process, with the needle aiming the vertebral arch between the spinous and the articular process. The needle was advanced in a slightly ventral, cranial, and medial direction. When the needle reached the lamina, it was withdrawn and then advanced again in a more cephalad angulation until the hard-elastic consistency of the ligamentum flavum between the T12 and T13 could be felt with the tip of the needle. The stylet was removed and an air-filled loss of resistance (LOR) syringe (Perifix; B. Braun) was connected to the needle. While advancing the needle, the operator pressed the syringe plunger until a LOR to air injection and a sudden LOR to needle advancement were felt. The procedure was aborted after the fourth attempt and recorded as a procedural failure. A plain solution of ropivacaine (5 mg/mL; Ropivacaina, Galenica senese, Italy) at 1 mg/kg and morphine (10 mg/mL; Morfina Cloridrato, Monico, Italy) at 0.1 mg/kg was slowly administered.

### Intrathecal analgesia

An intrathecal injection was administered via paramedian approach at the intervertebral space between L_5_ and L_6_ with the dog positioned as previously described. Four attempts to reach the subarachnoid space could be made using a 25G Quincke needle 50 to 75 mm in length (Aghi spinali; Pic, Italy). When the cerebrospinal fluid (CSF) outflow became visible in the hub of the needle, 30 μg/kg of preservative-free morphine diluted with saline solution to 0.6 mL total volume was injected over 20 to 40 s.

Anaesthesia was monitored by a clinician unaware of which block had been performed. During surgical access to the thoracic or the abdominal cavity, a bolus of fentanyl (1 μg/kg IV) was used as intraoperative rescue analgesia (iRA) if the HR and the MAP rose by more than 30% of the pre-incisional level, defined as the mean pressure measured during the 5 min before skin incision [15]. The fentanyl bolus was repeated every 3 min until the MAP reached the pre-incisional level. The incidence of iRA, the number of fentanyl boluses, and the time to first bolus were recorded for each group. Events of bradycardia (HR <60 beats/min) and hypotension (MAP <60 mm Hg for at least 5 min or MAP <55 mm Hg) were recorded. Hypotension was treated by reducing the sevoflurane end-tidal concentration by 0.2% and giving a 3 mL/kg bolus of lactated Ringer's solution IV in 60 s. An additional 2 mL/kg of fluid in 60 s was administered if the MAP was increased after the first bolus. If hypotension persisted, a bolus of ephedrine (50–100 μg/kg) and/or a continuous rate infusion (CRI) of norepinephrine (0.1–0.3 μg/kg/min) was given. When surgical access to the body cavity was achieved, the anaesthetists were free to use fentanyl as deemed useful to stabilize the anaesthetic plane. The amount of fentanyl administered during the procedure was recorded.

Body temperature was maintained with an active heating system (Bair Hugger Warmer Model 505; Augustine Biomedical Design, USA) during the perioperative period. The urinary bladder was voided manually at the end of the operation.

Quality of recovery from general anaesthesia was scored using a purpose-made scoring system (Additional file 1) [16]. Temperature, HR, RR, postoperative pain, and ability to walk were assessed at 30 min, 1, 2, 4, 6, 8, and 12 h after tracheal extubation by veterinarians trained in the use of the Short Form of the Glasgow Composite Pain Scale (SF-GCPS) [17] and blind to the treatment. Methadone 0.2 mg/kg was administered IM if the pain score was ≥ 6/24 or if two consecutive scores were 5/24. The reason for rescue analgesia administration was documented. The surgical incision was covered with an adhesive dressing (Primapore, Smith & Nephew, UK). After the last pain evaluation at T12, the study was considered completed. However, all the dogs were assessed every six hours using SF-GCPS. If necessary, methadone and meloxicam were given as rescue analgesia till 48 hours from surgery. Time from end of surgery to first postoperative methadone administration was recorded as was the number of boluses administered. Time to first feeding was recorded. Samples for arterial blood gas analysis (ABG) were obtained at 1 and 4 h after tracheal extubation. When the tests indicated PaO2 <80 mm Hg, oxygen supplementation was established via facial mask, flow-by or nasal cannula. Unexpected neurological sequelae were recorded up to 72 h after surgery.

### Statistical Analysis

Categorical variables are reported as frequency and percentage; Fisher’s exact test was used to evaluate frequency distribution independence between the two groups. The Lilliefors test was performed on continuous variables to check for normal distribution. Not normally distributed data are reported as the median and the range (minimum-maximum) and were analysed using the Mann–Whitney *U* test. Significance was set at 5% for all statistical methods.

## Results

A total of 40 dogs were initially enrolled. One TEA group dog was excluded because of severe intraoperative hemodynamic instability and another because of intraoperative fatal haemorrhage. Two ITM group dogs were excluded because subarachnoid puncture could not be performed. Demographics, type of surgery, and neoplasia were similar between the two groups (Table 1), as were median propofol induction bolus, time to the locoregional technique, time between local anaesthetic (LA) injection and skin incision, time between LA injection and the end of surgery, total anaesthesia duration, number of attempts and failures (Table 2). The amount and the number of boluses of crystalloids were significantly higher in the TEA group (*p=*0.029; *p=*0.043). Occurrence of bradycardia and hypotension, median HR and MAP, ephedrine consumption (doses and incidence) were similar between the two groups. The number of boluses of crystalloids administered were significantly higher in the TEA group (*p=*0.029; *p=*0.043) (Table 2).

**Table 1:** Demographics, type of surgery and neoplasia in the TEA (thoracic epidural anaesthesia) and the ITM (intrathecal morphine) group

	TEA (*n*=18)	ITM (*n*=18)	*P-value*
**Age (years)**	10 (1-14)	9.5 (5-14)	0.960
**Weight (kg)**	14 (5.5-46)	17 (5.5-42)	0.740
**Breed**	10 Mixed breed	5 Mixed breed	
	2 Dobermann	2 Shi-tzu	
	1 Labrador Retriever	2 German Shepard	
	1 Jack Russel Terrier	1 Bernese mountain dog	
	1 Lagotto romagnolo	1 Beagle	
	1Dachshund	1 American Staffordshire Terrier	
	1 Flat Coated Retriever	1 English Setter	
	1 Giant Schnauzer	1 Boxer	
		1 Yorkshire Terrier	
		1 Chinese Crested Dog	
		1 Epagneul breton	
		1 Springer Spaniel	
**Thoracic surgery (%)**	9/18 (50)	9/18 (50)	1
Thoracotomy for neoplasia	4/9	5/9	1
Sternotomy	2/9	3/9	1
Costectomy	2/9	1/9	1
Diaphragmatic hernia	1/9	0	1
**Abdominal surgeries:**	9/18 (50)	9/18 (50)	1
Gastrectomy	1/9	0	1
Hepatic lobectomy	3/9	2/9	1
Adrenalectomy	2/9	1/9	1
Pheochromocytoma resection	1/9	0	1
Splenectomy	2/9	3/9	1
Nephrectomy	0	1/9	1
Insulinoma resection	0	2/9	0.470
**Neoplasia:**			
Adenocarcinoma	8/18	2/18	0.059
Carcinoma	4/18	5/18	1
Chondrosarcoma	1/18	1/18	1
Thymoma	2/18	3/18	1
Pheochromocytoma	2/18	0	0.485
Histiocytic neoplasia	0	1/18	1
Sarcoma	0	2/18	0.485
Non neoplastic disease	1/18	4/18	0.033

**Table 2:** Procedural data, incidence of bradycardia and hypotension, median HR and MAP, ephedrine and crystalloids consumption (doses and incidence) of the TEA (thoracic epidural anaesthesia) and the ITM (intrathecal morphine) group. LA, local anaesthetic; min, minutes; n, number; HR, heart beats; MAP, mean arterial pressure

	TEA (*n*=18)	ITM (*n*=18)	*P-value*
Median propofol induction bolus (mg/kg)	5.05 (2.6-10)	5 (3.3-10)	0.54
Median time to perform the locoregional technique (min)	3.5 (1-15)	4 (1-7)	0.82
Attempts (n)	1 (1-4)	1 (1-2)	0.49
Failures (n)	0/18 (0)	2/18 (11.1)	0.48
Median time between LA injection and skin incision (min)	20 (8-40)	20 (10-87)	0.77
Median time between LA injection and the end of surgery (min)	148 (68-250)	132 (71-200)	0.76
Median time of entire anaesthesia duration (min)	234 (132-307)	196 (120-260)	0.17
Bradycardia (%)	1/18 (5.5)	3/18 (16.6)	0.60
Hypotension (%)	10/18 (55.5)	7/18 (38.8)	0.50
Median HR (beats/min)	90 (59-111)	82 (68-110)	0.87
Median MAP (mmHg)	72 (62-86)	73 (59-87)	0.27
Ephedrine (%)	2/17 (11.7)	6/18 (33.3)	0.22
Median ephedrine (γ/kg)	0 (0-130)	0 (0-50)	0.12
Boluses of crystalloids (%)	12/18 (66.6)	5/18 (27.7)	**0.04**
Median crystalloids (mL/kg)	3 (0-10)	0 (0-10)	**0.03**

The groups were similar for time to first meal and ambulation, median body temperature, itching, urinary retention, sedation (Table 3).

**Table 3:** Time to first meal, ambulation, median body temperature, itching, urinary retention, prolonged sedation in the TEA (thoracic epidural anaesthesia) and the ITM (intrathecal morphine) group

	TEA (*n*=18)	ITM (*n*=18)	*P-value*
Itching (%)	0	0	1
Urinary retention (%)	3/18 (16.6)	2/18 (11.1)	1
Sedation (%)	0	2/18 (11.1)	0.48
First meal (h since extubation)	8 (5-28)	17 (4-24)	0.45
Time to ambulation (h since extubation)	6 (4-16)	4 (2-12)	0.10
Temperature till 24 h (°C)	36 (35.9-38.2)	(36.5-38.5)	0.87

**Fig. 2:**
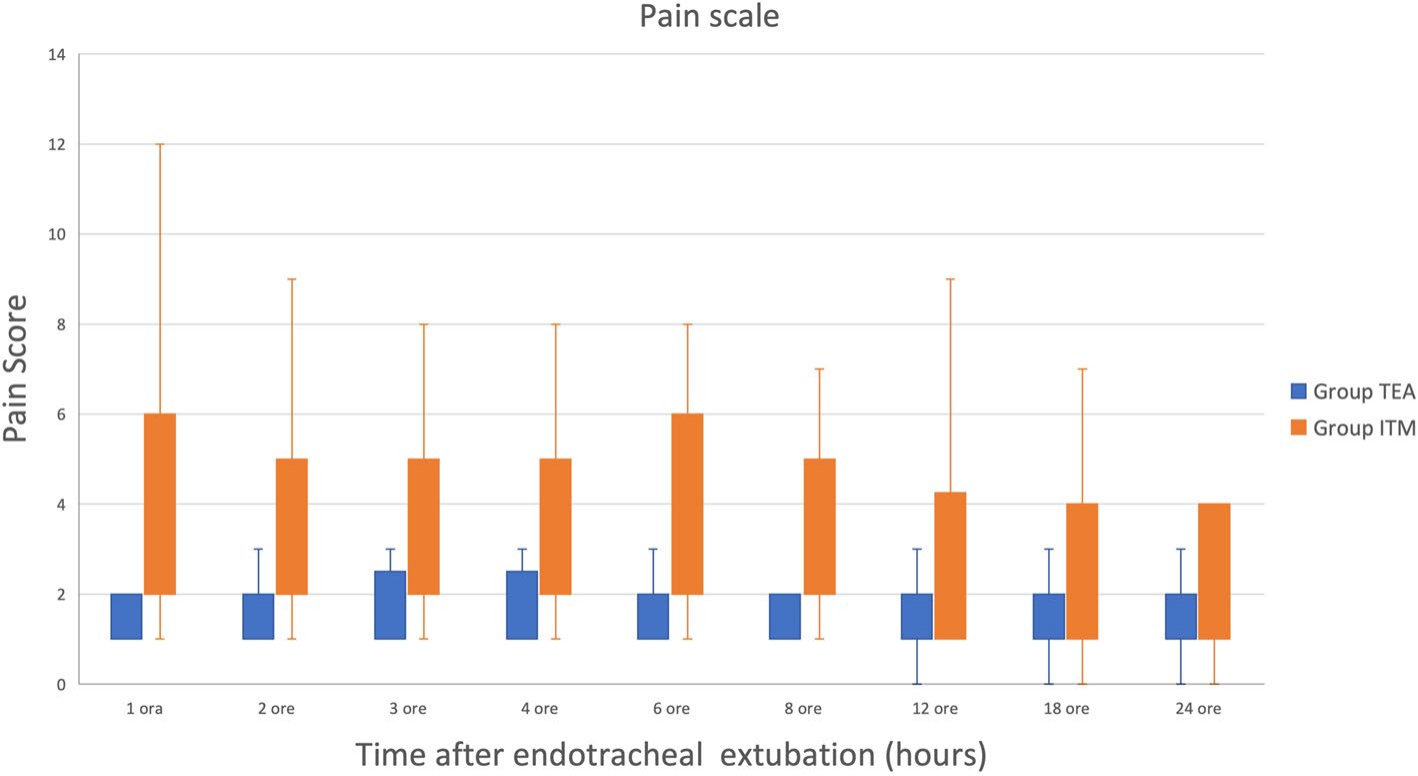
Pain scores (median and range) assessed using the short form glasgow compositive pain scale in the first 24 postoperative hours in dogs undergoing major thoracic and abdominal surgery in which TEA (thoracic epidural anaesthesia) and ITM (intrathecal morphine) was used to provide intraoperative analgesia

There was a statistically significant difference in iRA, median time to first fentanyl bolus, median fentanyl dose after surgical opening, and median GPS score at 30 min, 1 ,2, 4, 6, and 8 h between the groups (*p=*0.0003; *p=*0.0009; *p*<0.00001; *p=*0.002; *p=*0.005; *p=*0.0003; *p=*0.002; *p=*0.011; *p=*0.011) (Table 4; Fig. 2).

**Table 4:** Characteristics of the nociceptive and the analgesic block in the TEA (thoracic epidural anaesthesia) and the ITM (intrathecal morphine) group. iRA, intraoperative rescue analgesia; min, minutes; pRA, postoperative rescue analgesia; n, number; GPS, Glasgow pain score; T, time

	TEA (*n*=18)	ITM (*n*=18)	*P-value*
iRA (%)	8/18 (44.4)	18/18 (100)	**<0.001**
Median time to first fentanyl bolus (min)	50 (15-120)	7.5 (1-85)	**<0.001**
Median fentanyl after surgical opening (μg /kg)	0 (0-3)	7 (0-31.1)	**< 0.001**
pRA (%)	2/18 (11.1)	10/18 (55.5)	**0.01**
Total methadone administrations (n)	3	19	
GPS T1h	1 (1-7)	4 (1-12)	**<0.01**
GPS T2h	1 (1-4)	3 (1-9)	**<0.001**
GPS T4h	1 (1-5)	3 (1-8)	**<0.001**
GPS T6h	1 (1-5)	2 (1-8)	**0.01**
GPS T8h	1 (1-4)	2 (1-7)	**0.01**
GPS T12h	1 (0-3)	2 (1-9)	0.31
GPS T24h	1 (0-4)	2 (0-7)	0.08

There were significant differences in iRA, median time to first fentanyl bolus; median fentanyl dose after surgical opening, and median GPS score at 30 min, 1 ,2, 4, 6, and 8 h between the groups (*p<*0.001; *p<*0.001; *P* <0.001; *p<*0.001; *p<*0.001; *p<*0.001; *p<*0.001; *p<*0.01; *p<*0.01).

One TEA group dog (5%) required a bolus of fentanyl during surgical opening; none received intraoperative fentanyl CRI, whereas 15/18 (83%) ITM group dogs needed a total of 26 boluses of fentanyl and 15/18 (83%) received fentanyl CRI during surgery. Postoperative RA was required by 2/18 (11%) TEA and 10/18 (55%) ITM group dogs.

Urinary retention was recorded in 3/18 (16%) TEA and in 2/18 (11%) ITM group dogs. Time to ambulation was similar for both groups. Two ITM group dogs (11%) developed postoperative prolonged sedation lasting 6 and 24 h, respectively. Hypoxia and hypercapnia were revealed on the T2 blood gas analysis in two ITM group dogs undergoing sternotomy; both received oxygen supplementation until the second blood (T4) showed no abnormalities. Hypercapnia at T4 after splenectomy in one dog resolved within 2 h. No unexpected neurological sequelae were recorded.

## Discussion

Our data show that a single shot TEA with ropivacaine and morphine provided better control of nociceptive stimuli during surgical access to the thorax and the abdomen than intrathecal morphine. In TEA group only one dog needed iRA during surgical access to the thoracic or the abdominal wall, and 10/18 (55%) needed no fentanyl during the entire surgical procedure. Eight out of these ten (44%) dogs required no rescue opioid for 24 h.

The paramedian approach to the epidural space with the LOR technique facilitated correct needle positioning. Epidural anaesthesia is burdened by a high procedural failure rate in humans and in dogs [18, 19]. Although correct needle positioning was not confirmed by imaging, a postoperative transitory motor block of the hind limbs was noted in all TEA group dogs. Since the diameter of the thoracic spinal cord is smaller than that of the lumbar tract due to the intumescence, one can hypothesize that this facilitates even and complete distribution of LA around the spinal cord, thus improving the quality of block. In addition, the LA spreads equally cranially and caudally from the injection site at the thoracic spinal level [20], allowing for 0.2 mL/kg administered at the T_12_-T_13_ to provide an adequate level of perioperative analgesia.

Needle positioning is easier to carry out at the thoracolumbar spinal tract than at the lumbosacral tract because of the difference in length of the spinous processes and because the articular process can be palpated there. For this study we usually performed epidural puncture with a 20 G Tuohy needle (5 cm in length), which is easier to handle than longer needles. Also, unlike the median approach, needle positioning in the epidural space via the paramedian approach is guided by bone landmarks that help the anaesthetist direct the needle toward the epidural space. Loss of resistance was perceived in all TEA group dogs; the median number of attempts was one.

Most previous studies on TEA in dogs evaluated LA administration via an epidural catheter, however, the single shot TEA technique seems a rational option in many clinical situations because it provides 24 h of good analgesia, obviates potential catheter line complications (e.g., insertion, placement, removal, skin tunnelling, which is often necessary to fix the line on very mobile skin) and relieves ward staff of catheter management during hospitalization. Nevertheless, many dogs may have benefited from having an epidural catheter for better analgesia at 24 h after surgery.

The effect of TEA was compared with spinal morphine at 30 μg/kg. To the best of our knowledge, this is the first study to use intrathecal morphine as perioperative analgesia in dogs undergoing soft tissue surgery. Previous studies investigating the analgesic effect of epidural morphine in dogs undergoing thoracotomy found a good degree of postoperative analgesia [2, 21]. According to the literature, the amount of morphine reaching the CSF administered in the epidural space is between 2% and 4% of the total amount injected [22, 23]. In the present study, the amount of morphine reaching the CSF by direct spinal injection could have been more than ten times greater compared to standard dosing of epidural morphine. Although the ITM group dogs required rescue analgesia much more often than the TEA group, just over 50% required methadone during the postoperative period. Considering the high degree of pain expected in this group of dogs, the result is remarkable.

Intraoperative RA during thoracic or abdominal wall surgical access was administered earlier and more frequently in the ITM group dogs. Intrathecal morphine is of long duration but has a slow onset of action, with a peak effect at 8 h after administration [24]. For these reasons ITM was not expected to control nociceptive stimuli from surgical access, even though it is known to reduce the need for rescue analgesia in dogs undergoing hemilaminectomy [25].

After surgical opening, it was left to the discretion of the anaesthetists to administer fentanyl if they thought it would enhance stability of anaesthesia. Intra-operative analgesia was evaluated according to the number of iRA based on the increase in HR or MAP over the pre-incisional levels. This way to assess nociceptive reaction to surgical stimuli has proved useful for evaluating the quality of some regional anaesthesia block techniques in canine orthopaedic patients [26] because surgery produces cardiovascular changes through the somatic nociceptive pathway. We adopted this model to test the nervous block during surgical access to the thoracic or the abdominal cavity. During a thoracotomy or surgery involving the cranial abdomen, once surgical access to the cavity has been achieved, viscera manipulation, compression, or traction of the heart or of the great vessels, and surgeon interference with lung ventilation can produce cardiovascular variations independent of nociceptive stimuli. However, the extensive thoraco-abdominal sympathetic chain block produced by TEA may have attenuated sympathetic activation due to viscera manipulation, resulting in a more stable anaesthesia [27, 28]. This may explain the lower use of intraoperative fentanyl after surgical access to the body cavity was made.

The incidence of hypotension was similar for both groups; however, the TEA group received a greater amount of fluid and more ephedrine (about double the amount), though the difference was not statistically significant. TEA is known to cause sympathetic denervation, an alteration in the balance of sympathetic and parasympathetic activity, altered distribution of blood in relation to cardiac filling, and changes in myocardial function. Such changes can happen to a different degree, making the effects of TEA on the cardiovascular system complex and variable [29].

Two ITM group dogs developed postoperative prolonged sedation lasting 6 and 24 h, respectively. This is the first report of this side effect caused by intrathecally administered morphine in dogs. The prolonged permanence of hydrophilic morphine in the CSF allows it to spread cranially to the cerebrum and cause sedation. The morphine dosage we administered has been used in previous studies for many various surgical procedures, mainly for controlling perioperative orthopaedic pain [30–32] which reported no postoperative sedation. It is difficult to explain why postoperative prolonged sedation occurred in about 10% of cases. The dogs were neither particularly old nor hypothermic during recovery and their anaesthesia time was in one case above and in the other below the average of the ITM group. One dog received 25 μg/kg of fentanyl during prolonged surgery (140 min), which may have resulted in a certain degree of accumulation, given that intrathecal morphine has been reported to reduce the need for fentanyl [25]. Nevertheless, more data on spinal analgesia in dogs are needed, considering that postoperative monitoring in previous studies was short and that the dogs were adult animals undergoing orthopaedic surgery [30–32]. Ours is the first study to report on the use of intrathecal morphine in old dogs with cancer.

The present study has limitations. First, the study sample varied in age, size, type of and reason for surgery. Notwithstanding a certain degree of variability, our data well represent an average population undergoing thoracotomy or cranial celiotomy. Second, the lack of a strict anaesthesia protocol after surgical access warrants caution in interpreting the intraoperative data. Finally, the postoperative evaluations were conducted by different operators, all of which clinically experienced and blind to treatment.

## Conclusion

A single shot of TEA and ITM was effective in managing perioperative pain in elderly dogs with cancer undergoing major thoracic or cranial abdominal surgery. Both neuraxial techniques can be recommended owing to their remarkable perioperative analgesia, stable hemodynamics, good anaesthesia recovery, and postoperative opioid sparing effect. Further studies are needed to better investigate the real risk/benefit ratio of TEA and ITM in dogs.

## Supplementary Information

**Additional file 1.**

## Data Availability

The datasets generated and/or analysed during the current study are not publicly available due to privacy but are available from the corresponding author (EL) on reasonable request.

## Abbreviations

TEA: Thoracic epidural anaesthesia
ITM: Intrathecal morphine
IRA: Intraoperative rescue anaesthesia
GCPS: Glasgow Composite Pain Scale
pRA: Postoperative rescue analgesia
min: Minutes
h: Hours
CONSORT: Consolidated Standards of Reporting Trials
ASA: American Society of Anaesthesiologists
ACVIM: American College of Veterinary Internal Medicine
IM: Intramuscular
IV: Intravenously
FiO_2_: Inspired fraction of oxygen
P_E_’CO_2_: End tidal carbon dioxide pressure
HR: Heart rate
SAP; DAP; MAP: Systolic, diastolic, mean arterial pressures
RR: Respiratory rate
F_E_’_ISO_: End tidal isoflurane fraction
LOR: Loss of resistance
CSF: Cerebrospinal fluid
CRI: Continuous rate infusion
ABG: Arterial blood gas analysis
LA: Local anaesthetic
