# Supplementary material for: Thoracic epidural anaesthesia vs intrathecal morphine in dogs undergoing major thoracic and abdominal surgery: clinical study
Source: BMC Vet Res. 2022 May 27;18:200. doi: 10.1186/s12917-022-03296-3 (PMC9137148; doi:10.1186/s12917-022-03296-3)
Supplement: Supplementary file 1 — Additional file 1. [file 12917_2022_3296_MOESM1_ESM.docx]

**Appendix 1 –** Recovery score from the time of extubation to the first 15 minutes (modified from Liao et al., 2017).

0 Profound sedation (quiet, very relaxed, no restraint necessary for lateral recumbency, does not respond to voice or touch).

1 Very calm, with moderate sedation (quiet, relaxed, minimal restraint required for lateral recumbency, mild response to voice or touch).

2 Calm, however, minimal sedation (quiet but still alert and aware of surroundings, mild resistance to restraint for lateral recumbency, moderate response to voice and touch).

3 Minimal or no apparent sedation and/or excitable-dysphoric (excited, anxious, difficult to restraint in recumbency, very interactive and responsive, vocalizing, reactive to voice and touch).
